# Hospital Note

**Published:** 1896-06

**Authors:** 


### Hospital Note.

The second commencement of Riverside hospital took place Wednesday, May 27, 1896, one graduate, Margaret Johnston, receiving her diploma. The program consisted of addresses by Rev. Henry Elliott Mott and Rev. Thomas Calvert, with musical selections. The training class of 1897 consists of five pupils.

